# Functional Characterization of a Magnesium Transporter of Root Endophytic Fungus *Piriformospora indica*

**DOI:** 10.3389/fmicb.2018.03231

**Published:** 2019-01-09

**Authors:** Durga Prasad, Nidhi Verma, Madhunita Bakshi, Om Prakash Narayan, Alok Kumar Singh, Meenakshi Dua, Atul Kumar Johri

**Affiliations:** ^1^School of Life Sciences, Jawaharlal Nehru University, New Delhi, India; ^2^School of Environmental Sciences, Jawaharlal Nehru University, New Delhi, India

**Keywords:** *Piriformospora indica*, magnesium transporter, *Arabidopsis thaliana*, high affinity transporter, plant growth activity

## Abstract

Magnesium (Mg) is a crucial macronutrient required for the regular growth of plants. Here we report the identification, isolation and functional characterization of Mg-transporter *PiMgT1* in root endophytic fungus *Piriformospora indica*. We also report the role of *P. indica* in the improvement of the Mg nutrition of the plant particularly under Mg deficiency condition. Protein BLAST (BLASTp) for conserved domains analysis showed that PiMgT1 belong to CorA like protein family of bacteria. We have also observed the presence of conserved ‘GMN’ signature sequence which suggests that PiMgT1 belongs to Mg transporter family. Phylogenetic analysis revealed that PiMgT1 clustered among fungal CorA family members nearer to basidiomycetes. Functionality of *PiMgT1* was confirmed by complementation of a yeast magnesium transporter mutant CM66. We have observed that PiMgT1 restored the growth of mutant and showed comparable growth with that of WT. We found statistically significant (*p* < 0.05) two fold increase in the total intracellular Mg content of mutant complemented with *PiMgT1* as compared to the mutant. These observations suggest that PiMgT1 is actively involved in Mg uptake by the fungus and may be helping in the nutritional status of the host plant.

## Introduction

*Piriformospora indica* is a root endophyte isolated from the Thar desert of Rajasthan, India (Verma et al., 1998). Unlike arbuscular mycorrhizal fungi (AMF), *P. indica* can be cultured axenically and now a stable transformation system is available to know the gene functions in this fungus (Pham et al., 2008; Yadav et al., 2010). Further, *P. indica* has numerous beneficial effects on their host plant and help them to develop resistance against biotic as well as abiotic stresses (Waller et al., 2005; Baltruschat et al., 2008; Sherameti et al., 2008; Kumar et al., 2009; Vadassery et al., 2009; Sun et al., 2010; Yadav et al., 2010; Jogawat et al., 2013; Narayan et al., 2017).

It has been established that nutrient availability in the soil has major impact on the plant productivity. Metal ions are very important for various cellular and biochemical process as they play a vital role in various important biological processes. Mg is one of the most abundant elements on the earth and is an essential element which plays an important role in wide variety of biological processes. Being, central atom of the chlorophyll molecule, Mg plays a huge role in photosynthesis in plants. Additionally, Mg also acts as a cofactor in various biological enzymatic activities like RNA polymerase, DNA polymerase, ATPase, protein kinases, phosphatases and other cellular processes (Williams, 1993; Elin, 1994; Cowan, 1995; Shaul, 2002; Gardner, 2003).

Mg deficiency is an unfavorable plant disorder that occurs most often in strongly acidic, light, sandy soils, where it can easily leak away. It is a crucial macronutrient having dry mass of 0.2–0.4% and is compulsory for regular growth of plant. Due to deficiency of Mg, plants start damaging the chlorophyll in the old leaves which results in the yellowing of leaves. This condition is known as chlorosis which imparts the mottled appearance of leaves. In older leaves due to prolonged Mg deficiencies necrosis and drooping takes place.

Arbuscular mycorrhizal fungi has been reported to play important role in the uptake of the Mg from the soil and helps the plant grow healthy under Mg deficiency conditions (Wu et al., 2006; Doubková et al., 2012; Zhang et al., 2015). However, by taking a closer look of these reports, we found that no information is available on the functional characterization of the Mg transporter which is involved in the Mg transporter activity of the AMF. In the present work we have identified and functionally characterized a Mg transporter of *P. indica*. We recommend that utilization of *P. indica* and its Mg transporter not only complement crop-growing approaches, but may also be used as a model system to study the molecular mechanism of symbiotic plant–microbe interaction.

## Materials and Methods

### Maintenance of Bacterial, Fungal, and Plant Culture

Luria Bertani agar plates containing adequate amount of ampicillin (antibiotic) were used to maintain the bacterial strains. *P. indica* was cultured regularly on solidified *Aspergillus* modified medium (AMM) (Hill and Kafer, 2001) and incubated at 30 ± 2°C for 8–10 days. *P. indica* was also cultured in AMM broth in 250 ml culture flasks at 30 ± 2°C, and 110 rpm in shaking incubator (Multitron Incubator Shaker, HT-Infors, Switzerland). The yeast strains were maintained on YPD and SD medium at 30 + 2°C for 72 h. *A. thaliana* plants were maintained in controlled glasshouse conditions with a 10-h light (1000 Lux)/14-h dark period at temperature of 25 ± 2°C with a relative humidity 60–70%.

### Interaction of *A. thaliana* and *P. indica*

*Arabidopsis thaliana* WT (ecotype Columbia-0) seeds were surface sterilized for 2 min in 0.1% mercuric chloride (HgCl_2_) and ethanol followed by 10 min in NaClO solution (0.75% Cl) and finally washed six times with sterile water and placed on Petri dishes containing MS medium (Murashige and Skoog, 1962). After cold treatment for 48 h at 4°C, plates were incubated for 10 days at 22°C under illumination (100 μmol m^-2^ s^-1^). *P. indica* was cultured as mentioned previously on AMM (Yadav et al., 2010). Co-cultivation of *A. thaliana* with the fungus *P. indica* was performed under *in vitro* culture conditions on solidified PNM media (Johnson et al., 2011). To do this, one *P. indica* disk was cut with the help of inverted portion of 1 ml tip and placed at the center on each plate and were allowed to grow for 10 days in dark. In case of plant, seedlings were first given cold treatment (4°C) for 48 h. After this treatment seedlings were allowed to grow for 10 days. Further, equal size seedlings were used for the co-cultivation assay. In order to study impact of Mg on plant growth and development during colonization with *P. indica*, plants colonized with *P. indica* were grown on PNM media and treated with five different Mg concentrations, i.e., 0, 2, 4, 10, and 25 mM. Seedlings were allowed to grow for 15 days at 22°C and 70–80% humidity in a 16-h light/8-h dark cycle. Plants were harvested after 15 dpi and total biomass, root length, number of leaves and chlorophyll content were measured. Kaefer media (KF) disk was used for mock treatment and mock-treated seedlings were used as control.

### Root Colonization

Roots of *A. thaliana* cultivated with and without *P. indica*, were harvested after 15th dpi, washed intensively with double distilled water. In order to check the presence of chlamydospore in the roots, histochemical analysis was performed. To study colonization ten root samples from *A. thaliana* plant were randomly selected. Softening of the samples was done using 10% KOH solution for 15 min. Further samples were acidified with 1N HCl for 15 min. Finally, staining was performed using 0.02% Trypan blue overnight (Phillips and Hayman, 1970; Dickson and Smith, 1998; Kumar et al., 2009). The samples were de-stained with 50% lacto-phenol for 1–2 h prior to observation under the light microscope (Leica Microscope, Type 020-518.500, Germany and Nikon Eclipse Ti). Distribution of intracellular chlamydospores within the cortex region of root was taken as a symptom of colonization (McGonigle et al., 1990; Kumar et al., 2009).

$$\begin{array}{c} \mathrm{Percent colonization}=\frac{\mathrm{Number of colonized roots}}{\mathrm{Total numbers of segments}}\times 100 \end{array}$$

### Determination of Total Biomass, Root Length and Leaves Number

To study the impact of different concentration of Mg in the presence and in absence of *P. indica* the plant total biomass, root length, and number of leaves of the inoculated and non-inoculated plant after 15 days was measured as described (Jogawat et al., 2013). To calculate the total plant biomass, plants were harvested from co-cultivation plate and were washed properly with ddH_2_O and plants surface moisture was removed by keeping them on blotting paper and then the weight was recorded.

### Measurement of Chlorophyll Content

To determine chlorophyll contents, leaves were harvested, weighed and was grounded in 90% ammonical acetone (acetone: water: 0.1 N ammonia, ratio of 90: 9: 1) at 4°C. This mixture was centrifuged at 12000 rpm at 4°C for 10 min. After centrifugation, supernatant obtained was used for the pigments measurement. Pigments contents were measured at 663 and 645 nm for Chl a, Chl b, respectively. Chlorophyll contents were calculated (as nmol/ml.), as follows, Chl a = (14.21 × OD663 - 3.01 × OD645), Chl b = (25.23 × OD645 - 5.16 × OD663), and total Chl (a+b) = (9.05 × OD663 + 22.2 × OD645). The obtained values were divided by leaf fresh weight to obtain values in nmol/ml/mg of leaf fresh weight as described (Porra et al., 1989).

### Identification of Putative Mg Transporter

To identify Mg transporter in *P. indica*, genome database of *P. indica* (Zuccaro et al., 2011) was investigated. For this purpose, ALR1p nucleotides sequence of *Saccharomyces cerevisiae* was used as query. For BLASTP, we have fixed a cut off value of *E* = 10^-2^. Further, *PiMgT1* and *PiMgT2* were identified based on the amino acid similarity and domain analysis. Nucleotide sequence was studied using bioinformatics approaches such as GeneScan^1^, Translate tool^2^, nucleotide BLAST (BLASTn) and BLASTx^3^.

### Isolation and Cloning of Putative Magnesium Transporter (*PiMgT1* and *PiMgT2*)

#### Isolation of RNA and cDNA Synthesis

To do this, *P. indica* was grown in Kaefer media (Hill and Kafer, 2001) and MN media (Bécard and Fortin, 1988). Further, *P. indica* was washed twice with autoclaved ddH_2_O and transferred to MN media supplemented with 10 μM MgCl_2_ as a sole source of Mg. Ten days old culture of *P. indica* was harvested, freeze in liquid Nitrogen and stored at -80°C.

Total RNA was isolated with TRIzol reagent following the protocol provided by the manufacturer (Invitrogen, United States). Approximately 0.8 g fungal tissue was grounded to fine powder. To this, 1 ml TRIzol reagent was added and this was mixed vigorously. The homogenized samples were incubated further for 15 min at RT. Further, 200 μl chloroform was added (per ml of TRIzol reagent used) and this mixture was vigorously vortexed for 30 s and incubated at RT for 10 min. Following centrifugation (13,000 rpm; 15′; 4°C), upper aqueous phase was transferred into fresh RNase free tube (pre-cooled). The RNA from the aqueous layer was precipitated by mixing with 0.7 volumes of isopropyl alcohol. This mixture was incubated for 1 h at 4°C and centrifuged (12,000 rpm; 10′; 4°C). RNA pellet obtained was washed twice with 75% ethanol, centrifuged (10,000 rpm for 5 min at 4°C). Finally, RNA pellet obtained was dried for 10 min and dissolved in adequate volume of DEPC-treated water and quantified (NanoDrop 1000 spectrophotometer, Thermo Scientific).

### Amplification of Putative *P. indica PiMgT1* and *PiMgT2*

Predicted putative *PiMgT1* and *PiMgT2* were PCR amplified by using gene specific primers (Supplementary Table S1). For PCR reaction, *P. indica* cDNA was used as a template. PCR reactions were carried out in a final volume of 50 μl, containing 10 mM Tris-HCl (pH 8.3); 50 mM KCl; 1.5 mM MgCl2; 200 μM of dNTPs; 3 μM of each primer; 3 units of DNA *Pfu*-polymerase and 60–100 ng of cDNA as template. PCR program was used as follows: 94°C for 2 min (1 cycle), 94°C for 45 s, 59°C for 1 min 15 s, 72°C for 2.5 min (35 cycles) 72°C for 5 min (1 cycle), 4°C to hold the reaction. Amplified DNA was digested with specific restriction enzymes. PiMgT1 was digested with *KPNI-XBAI* and PiMgT2 with *BamHI-XBAI*. Digested DNA was electrophoresed and eluted using MinElute gel extraction kit (Qiagen, Germany). Eluted DNA was stored at -20°C. For cloning, ligation was performed at 4°C, overnight and for this purpose 1:3 of the vector-insert molar ratio was used. For transformation, *Escherichia coli* DH5-α bacterial strains were made competent as described by Inoue et al. (1990). The ligated product or plasmid was directly added to 200 μl competent cell suspension, mixed and was kept on ice for 30 min. The cells were then given a heat shock at 42°C for 90 s and quickly chilled on ice for 5 min. This was followed by addition of 0.8 ml of LB, and cells were incubated at 37°C for 45 min. The transformed competent cells were plated on LB plate containing ampicillin (100 μg/ml) and were incubated at 37°C overnight. Individual colonies were picked from overnight grown culture and mixed in 10 μl sterile dH_2_O in a 0.5 ml microcentrifuge tube. The lysis of cells was done by boiling for 2 min. After centrifugation, supernatant (5 μl) was used as a template for PCR. Colony PCR was carried out to confirm the presence of the insert in the transformed clone by using gene-specific primers (Supplementary Table S1). Further cloning was confirmed by restriction digestion and sequencing (GCC Biotech, West Bengal, India).

### Phylogenetic and Homology Analysis

The functional sites and their pattern in putative PiMgT1 and PiMgT2 protein were determined using PROSITE data bank^4^. For identification purposes, BLASTn and BLASTx algorithm^5^ was used. Multalign analysis was done by using CLUSTALW software^6^. Phylogenetic and molecular evolutionary analyses of *P. indica* both putative *PiMgT1* and *PiMgT2* were conducted using MEGA7 with neighbor-joining (N-J) method^7^ (Tamura et al., 2011). Phylogenetic tree was constructed, with homologs protein of PiMgT1 and PiMgT2 from other fungi and other different groups like plant, bacteria and mammals (Supplementary Table S2).

### Complementation Assay

The yeast CM66 mutant (MATa alr1::HIS3alr2::TRP1 his3-Δ200, ura3-52, leu2-Δ1, lys2-Δ202 trp1-Δ63) was used for the complementation assay to check the functionality of the of putative PiMgT1 and PiMgT2 protein. CM66 mutant of yeast *S. cerevisiae* lacks the plasma membrane-localized Mg^2+^ transporter genes *ALR1* and *ALR2* (Li et al., 2001). Both the genes *PiMgT1* and *PiMgT2* was subcloned into pYES2 yeast expression vector and positive clones were transformed into CM66 mutant.

Yeast mutant was used for transformation as described (Gietz et al., 1995). A single colony was inoculated into 20 ml YPD and incubated at 30°C at 220 rpm until OD_650_ reaches 1–1.5. One ml of freshly grown culture was transferred to fresh 100 ml YPD medium to produce an initial OD_600_ of 0.5. After reaching the OD_600_ of 0.5, cells were pelleted down by centrifugation at 4000 rpm for 5 min at RT. Supernatant was discarded and cell pellet was re-suspended in 50 ml H_2_O. Cells were again centrifuged (4000 rpm, 5 min, RT), supernatant was discarded and pellet obtained was re-suspended in 0.5 ml sterile LiAc solution [1 ml 10 × LiAc (1 M LiAc pH 7.5), 1 ml 10 × TE (0.1 M Tris-HCl pH 7.5, 10 mM EDTA), 8 ml H_2_O]. 100 μl of cell suspension was dispended in 1.5 ml micro centrifuge tubes, and 0.1 μg of each type of plasmid was added together with 100 μg of salmon sperm DNA followed by addition of 0.6 ml sterile PEG/LiAc solution (8 ml 50% PEG, 1 ml 10 × TE, 1 ml 10 × LiAc) to each tube and vortexed. Micro centrifuge tubes were incubated at 30°C for 30 min at 250 rpm. Post incubation period, 70 μl of 100% DMSO was added (10% final conc.) and this mixture was mixed gently. Further, heat shock treatment was given for 15 min at 42°C, followed by chilling on ice for 5 min and later was centrifuged for 1 min at 13,000 rpm. After centrifugation supernatant was discarded and cell pellet obtained was re-suspended in 0.5 mL of TE buffer. Finally 100 μl of this mixture was placed on Petri plates containing the appropriate selection medium (SD-URA^-^). Plates were incubated for 3–4 days until colonies appear. Positive clones were confirmed by performing plasmid PCR and by sequencing.

### Functional Characterization

#### Growth Analysis

To study the growth pattern of *S. cerevisiae* WT, mutant transformed with vector only, and mutant transformed with PiMgT1 and PiMgT2 were inoculated separately in SD media supplemented with 250 mM MgCl_2_. Culture was allowed to grow till 0.6 OD_600_. This culture was harvested by centrifugation at 500 *g* for 2 min at 4°C. This culture was washed with 0.1 mM EDTA followed by washing with the autoclaved ddH_2_O. Equal number of cells (0.1 OD_600_) was taken for each strain and inoculated separately in SD media supplemented with different concentration of Mg, i.e., 0.1, 1, 4, 10, 25, and 100 mM. Culture was incubated at 30°C and OD_600_ were recorded at four hrs regular time intervals to observe comparison between the WT, mutant transformed with vector only and complemented mutants.

#### Spot Assay

To perform the spot assay *S. cerevisiae* WT, mutant transformed with vector only, mutant transformed with PiMgT1 and PiMgT2 were grown into YNB+URA^-^ medium containing 250 mM MgCl_2_. Culture pellet was harvested by centrifugation at 500 *g* for 2 min at 4°C. Each culture was washed with 0.9% sodium chloride solution followed by washing with autoclaved ddH_2_O. Each cultures was serially diluted starting with fixed 0.1 OD_600_ (10^-1^, 10^-2^, 10^-3^, and 10^-4^) and was spotted in a row on YNB deficient in URA^-^ agar plates supplemented with different concentration of MgCl_2_ (10 μM, 0.1 mM, 1, 4, 10, and 100 mM). The plates were incubated at 30°C for 5–10 days to observe comparison between the WT, complemented mutants and mutant transformed with vector only.

#### Cobalt Resistance Assay

To perform the cobalt resistance assay, *S. cerevisiae* WT, mutant transformed with vector, mutant transformed with PiMgT1 and PiMgT2 were cultured in YNB+URA^-^ medium containing 250 mM MgCl_2_. Culture was harvested by centrifugation at 500 *g* for 2 min at 4°C. Each culture was washed with 0.9% sodium chloride solution followed by washing with autoclaved ddH_2_O. Each culture was serially diluted starting with fixed 0.1 OD_600_ (10^-1^, 10^-2^, 10^-3^, and 10^-4^) and was spotted in a row on YNB deficient in URA^-^ agar plates supplemented with different concentration of CoCl_2_ (100 and 500 μM) and fixed concentration of MgCl_2_ (100 mM) in the medium. The plates were incubated at 30°C for 5–10 days to observe comparison between WT, complemented mutants and mutant transformed with vector only.

#### Uptake of Mg

To study uptake Mg, *S. cerevisiae* WT, mutant transformed with vector, mutant transformed with PiMgT1 were grown upto log phase (OD_600_ = 0.6 to 0.8) in LPM (low phosphate medium) medium containing 250 mM MgCl_2_. Cells were harvested by centrifugation at 500 *g* for 5 min at RT. Further, cells were washed with milli-Q water containing 1 mM EDTA and twice with autoclaved ddH_2_O and were incubated in LPM media without MgCl_2_ for 24 h. After starvation, culture was harvested by centrifugation at 500 *g* for 2 min. To energize, equal number of cells were re-suspended in 5 mM Tris-succinate buffer, at pH 4, supplemented with 1% galactose and incubated for 30 min at 25°C. To perform the uptake, yeast culture was centrifuged at 500 *g*, pellet obtained was re-suspended in uptake buffer (5 mM Tris-succinate buffer, pH 4, supplemented containing 4 mM MgCl_2_). Samples of 250 mL was withdrawn at different time points (0, 10, 20, 40, 60, and 120 min). To remove excess salts adhered with the cell surface, culture was washed three times with pre-chilled 30 mL of the washing solution (Milli-Q water, 1 mM EDTA). Further, pellet was collected by centrifugation at 500 *g* for 5 min. Pellet was air dried for 24–48 h and crushed with the help of mortar and pestle and fine powder was analyzed by EDXRF (Perring et al., 2017). An ED-XRF Epsilon 5XLE from PANalytical (Almelo, Netherlands) equipped with silver anode tube (maximum voltage = 50 kV) was used. Each sample was mixed with the 1 ml polymer binder to provide smooth surface. Further, settings for accelerating voltage and current were optimized in order to get the best compromise of sensitivity for the investigating Mg content regarding raw peak heights and ratios between raw peak and background heights. Mg content was estimated in the sample for 180 s. Zone of low energy with high overlapping emission lines, high resolution mode was preferred to better distinguish the energies of element Mg. Mg was analyzed at high resolution by setting background at region of interest (1.200) and region of maximum (1.280). Per pellet 8 min was devoted for total time of analysis, corresponding to the sum of measurement times and adjustment of flow of current, more time for atmosphere flushing, changing of filter, and holder rotation of sample holders. Three pellets were analyzed for each sample. Variability of investing yeast samples lead to matrix effects in calibration curves. Therefore, the background due to scattered primary radiation, was used to systematically correct all element ED-XRF signals. Calibration curves were established using the supplier software according to following formula:

Concentration = a × (Peak Count Rate = Background Count Rate) + b - overlapping factor × Peak count rate over lapping rate.

All pellets were analyzed and was mixed with the same polymer with similar ED-XRF conditions.

### Statistical Analyses

The significance difference between colonized with *P. indica* and non-colonized plants was determined by unpaired *t* testing. *P*-values of <0.05 were considered significant. These statistical analyses were performed with Instat (GraphPad, San Diego, CA, United States).

## Results

### Growth Promotion Activity by *P. indica*

**FIGURE 1:**
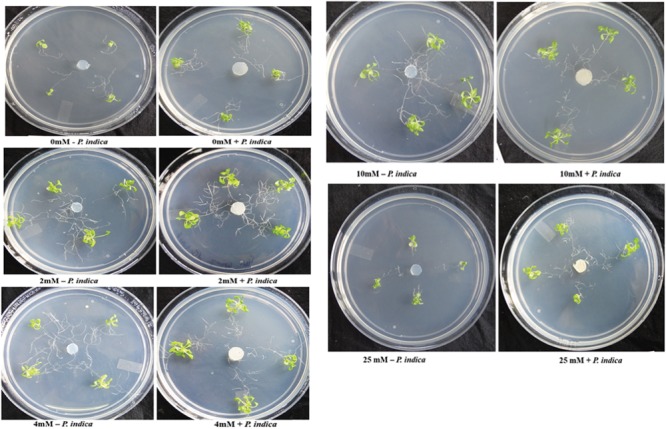
Interaction of plant with *Piriformospora indica*. Plants were cultivated with or without *P. indica* in PNM media supplemented with different concentration of Mg for 15 days under long day light conditions.

**FIGURE 2:**
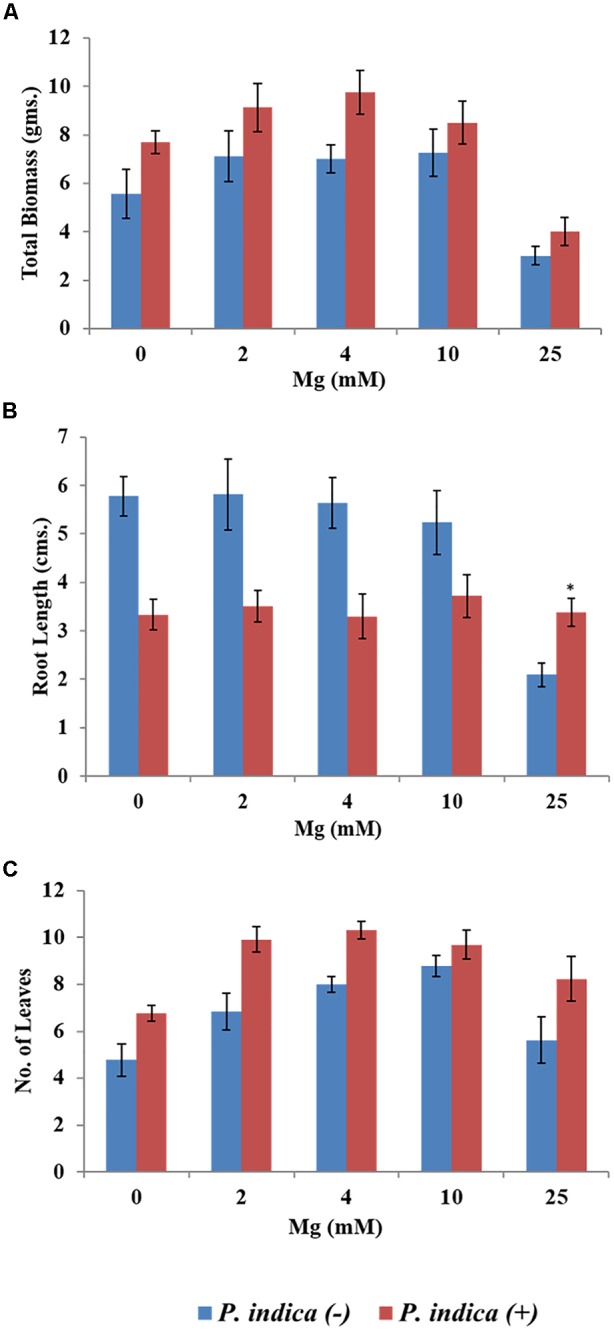
Impact of *P. indica* colonization on the growth of the plant. Growth parameters of Arabidopsis seedlings after 15 days of co-cultivation under different Mg concentration **(A)** Biomass **(B)** Root length **(C)** Number of leaves. Each data set represents the means of 3 independent measurements ± SE ^∗^indicates not significant as compared with the control (non-colonized); all other data are found significant at *P* < 0.05.

**FIGURE 3:**
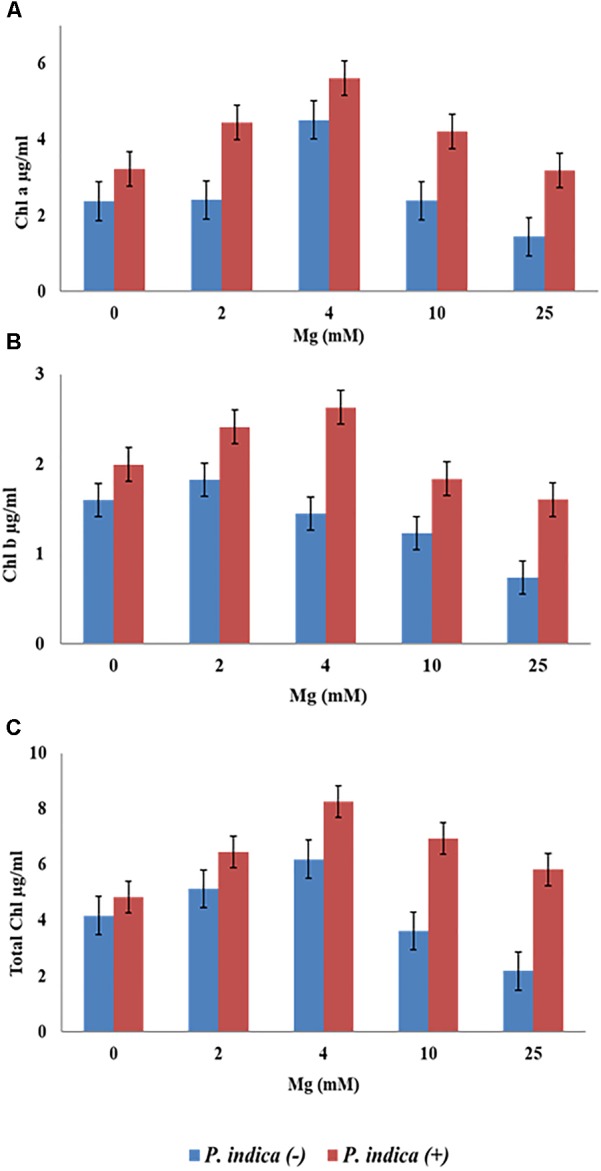
Measurement of chlorophyll contents of the plant during colonization with *P. indica* and during non-colonized stage. *A. thaliana* seedlings after 15 days of co-cultivation under different Mg concentration **(A)** chlorophyll a **(B)** chlorophyll b **(C)** total chlorophyll. Each data set represents the means of three independent measurements; all data are found significant at *P* < 0.05.

We have determined the growth of *A. thaliana* in the presence of different concentrations of Mg, i.e., 0, 2, 4, 10, and 25 mM during colonization with *P. indica* and non-colonized stage (Figure 1). Plants were found to be healthy when colonized with the *P. indica* as compared to the non-colonized plants at all the Mg concentrations (Figure 1). In order to test the impact of *P. indica* on plant growth and development, growth parameters like, total biomass, root length, number of leaves and chlorophyll contents were measured. Total plant biomass was found to be significantly high at 4 mM Mg when colonized with *P. indica* as compared to non-colonized plants (*P* = 0.03) (Figure 2A). Interestingly, biomass of the plants was found to be severely affected at 25 mM of Mg in both colonized and non-colonized state (Figure 2A). Root length of the non-colonized plants were found to be longer in comparison to *P. indica* colonized plants from 0 to 10 mM Mg concentration (Figures 1, 2B). However, in case of plants colonized with the *P*. *indica* and non-colonized plants treated with the high concentration of Mg, i.e., 25 mM, root length was found to be short in both the cases (Figures 1, 2B). The root of the non-colonized plants grown at a different concentrations of Mg (0–10 mM) were found very long along with numerous lateral roots, however, plants colonized with *P. indica* developed shorter roots as well as a lesser number of lateral roots (Figures 1, 2B). In case of colonized plants, leaves numbers were found to be high at all Mg concentrations as compared to the non-colonized plants (Figures 1, 2C). We have observed increase contents of chlorophyll a, b and total chlorophyll contents in plants colonized with the *P. indica* in comparison to the non-colonized plants at all Mg concentrations (Figures 3A–C). Additionally, chlorophyll contents was found to be less in the non-colonized and also in case of colonized plants at 0 mM Mg as compared to the colonized plant (Figures 3A–C). We have observed that level of colonization in the roots increased from 8 to 70% as the number of days progressed. A maximum percent of colonization, i.e., 70% was observed after 15 days of post inoculation of *P. indica* (Table 1).

**Table 1:** Percent colonization of *Piriformospora indica* in *Arabidopsis thaliana.*

Time after inoculation (days)	Percent colonization (%)
5	8 ± 4
10	27 ± 13.4
15	70 ± 14.6

### Identification and Isolation of Mg Transporter

In order to identify putative Mg transporter, BLASTn analysis was performed, by using the local alignment of the whole genome sequence of *P. indica* with the *S. cerevisiae* Mg transporter (ALR1p-1) as a query. Our analysis showed the presence of two homolog of ALR1p-1 named *PiMgT1* and *PiMgT2* in the genome of *P. indica*. We found that *PiMgT1* and *PiMgT2* belong to PIRI_contig_0025. Both the proteins PiMgT1 and PiMgT2 (accession number CCA67920.1 and CCA67912.1) shares 20 and 16% of query coverage as well as identity 31 and 25%, respectively, with the ALR1 protein of *S. cerevisiae* (Table 2). Protein BLAST (BLASTp) for conserved domains analysis showed that both putative transporters belong to CorA like protein family of bacteria.

**Table 2:** BLASTn analysis for putative *P. indica PiMgT1* and *PiMgT2* gene sequence of *P. indica* genome.

Description	Max score	Total score	Query cover	*E*-value (%)	Identity (%)	Accession
Related to Mg^2+^ transporter protein, CorA-like [*P. indica* DSM 11827]	43.1	73.1	20	3e-04	31	CCA67920.1
Related to Mg^2+^ transporter protein, CorA-like [*P. indica* DSM 11827]	33.5	66.6	16	0.23	25	CCA67912.1

*Result shows first hit in PIRI_contig_0025 as the most probable putative PiMgT1 and PiMgT2 gene. Table showing E-value and identity of sequences with yeast ALR1p as query sequence.*

### Cloning of Putative Mg Transporter *PiMgT1* and *PiMgT2*

After identification, both putative genes *PiMgT1* and *PiMgT2* ORF were cloned into a pGEMT-easy vector, sequenced and subsequently sub-cloned in pYES2 yeast shuttle vector. Positive clones were confirmed by colony PCR, restriction digestion and by sequencing. We found that putative *PiMgT1* and *PiMgT2* sequence was 1938 and 1923 bp long, respectively, with ATG as a start, TGA and TAG as stop codon, respectively (Supplementary Figures S1, S2).

### Phylogenetic and Homology Analysis

**FIGURE 4:**
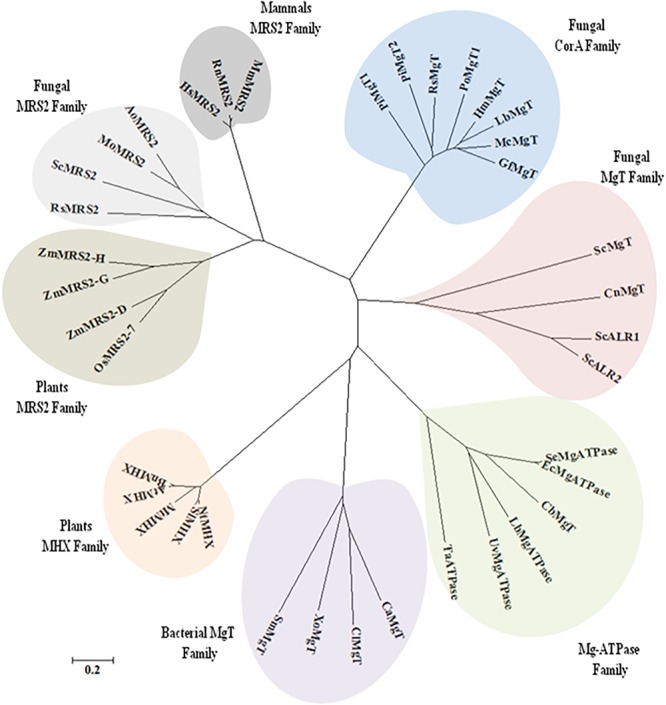
The phylogeny tree was inferred by using the maximum likelihood method based on the JTT matrix-based model. The tree with the highest log likelihood (–6494.5969) is shown. Boot strap value of 500 was taken in the tree construction. Initial tree(s) for the heuristic search were obtained automatically by applying Neighbor-Join and BioNJ algorithms to a matrix of pair wise distances estimated using a JTT model, and then selecting the topology with superior log likelihood value. The tree is drawn to scale, with branch lengths measured in the number of substitutions per site. The analysis involved 44 amino acid sequences. All positions containing gaps and missing data were eliminated. There were a total of 99 positions in the final dataset. Evolutionary analyses were conducted using MEGA7.

**FIGURE 5:**
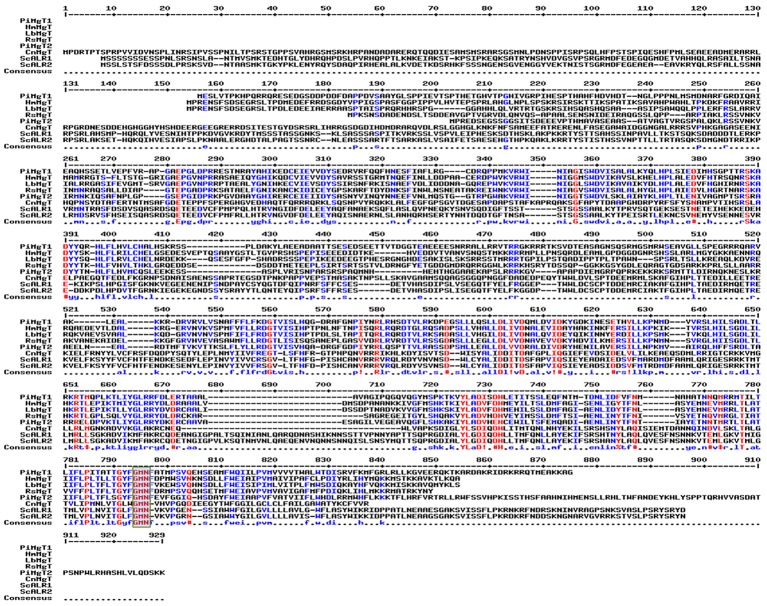
Multiple alignment analysis. Multiple alignments analysis of *PiMgT1* and *PiMgT2* was performed using MultAlign tool with six homologs from closely related fungi. The analysis revealed conserved ‘GMN’ signature sequence (highlighted in rectangular blue color box) which is required to be designated as an Mg transporter.

It was found that both the putative *PiMgT1* and *PiMgT2* has all important motif, domains, and sites which are important for a protein to be defined as a member of CorA family. Both the putative *PiMgT1* (361–598) and *PiMgT2* (293–546) bears CorA domain. In case of *PiMgT1* CorA domain was found to be conserved between 351 amino acid to 597 amino acid whereas, in case of *PiMgT2* CorA was found to be between 301 amino acid and 539 amino acid. Both Mg transporters were found to be clustered among fungal CorA family members nearer to basidiomycetes (Figure 4). Multiple alignments analysis of *PiMgT1* and *PiMgT2* was performed which revealed that both of them have conserved ‘GMN’ signature sequence which is required to be defined as a Mg transporter (Figure 5).

Our BLASTX analysis of putative *PiMgT1* demonstrated that putative Mg transporter has approximately 44% identity with Mg transporter of *Laccaria bicolor* from the division of basidiomycete. Whereas, it has 38% similarity with the plant *A. thaliana.* Further, we found that, this putative Mg transporter has 36% and 28% similarity with *Cyanobacterium aponinum* (bacteria) and *Homo sapiens*, respectively (Table 3).

**Table 3:** Summary of amino acid identity (%) between *P. indica* Mg transporter *PiMgT1* and other fungal, plant and animal magnesium transporters.

Organism	Mg transporter (Number of amino acids)	GenBank Accession number	Identity with PiMgT1 (%)	Group
*Piriformospora indica*	PiMgT1 (646)	CCA67920.1	100	Fungi
*Piriformospora. indica*	PiMgT2 (641)	CCA67912.1	42	Fungi
*Laccaria bicolor*	LbMgT (635)	XP_001874189.1	44	Fungi
*Saccharomyces cerevisiae*	ScALR1 (859)	Q08269.1	31	Yeast
*Saccharomyces cerevisiae*	ScALR2 (858)	P43553.1	28	Yeast
*Arabidopsis thaliana*	AtMHX (539)	AAF14229.1	38	Plant
*Cyanobacterium aponinum*	CaMgT (465)	AFZ52191.1	36	Bacteria
*Homo sapiens*	HmMRS2 (443)	NP_065713.1	28	Mammal

### Complementation Assay

#### PiMgT1 Functionally Complement Mg Transporter Mutant

**FIGURE 6:**
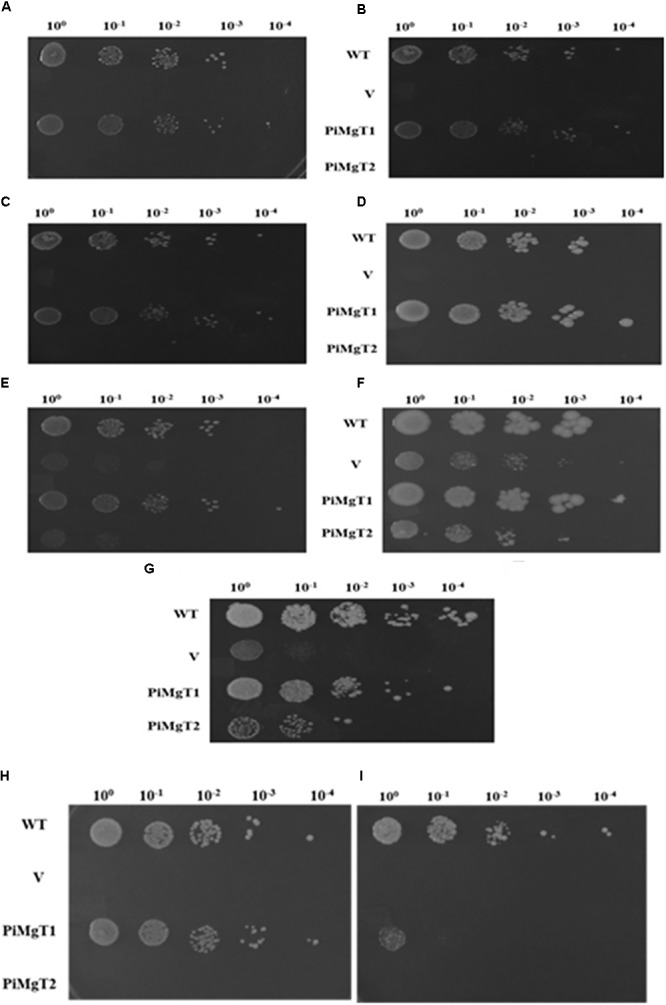
Complementation assay. Growth complementation assay in comparison of WT of the mutant *S. cerevisiae* strain CM66 transformed individually with plasmid vector only (V), PiMgT1 and PiMgT2 indicated and serially dilution of each strain was spotted on SC^-^URA medium containing different concentration of Mg **(A)** 10 μM **(B)** 100 μM **(C)** 1 mM **(D)** 4 mM **(E)** 10 mM **(F)** 100 mM **(G)** Control on YPD. Cobalt resistance assay: cobalt resistance assay in comparison of WT of the mutants *S. cerevisiae* strain CM66 which lacks Mg transport systems was transformed with plasmids vector only (V) PiMgT1 and PiMgT2 indicated and serially dilution of each strain was spotted on SC^-^URA medium containing different concentration of CoCl_2_ **(H)** 100 μM CoCl_2_ **(I)** 500 μM CoCl_2_ and fixed amount of 100 mM MgCl_2_.

We have analyzed functional expression of putative PiMgT1 and PiMgT2 in Mg transporter mutant. Complementation assay was performed using the yeast mutant CM66, which lacks *ALR1* and *ALR2* gene responsible for Mg transportation, therefore, cannot grow on standard medium containing less than 4 mM Mg^2+^. We have found that complemented mutant has restored good growth in case of PiMgT1 as compared to the PiMgT2. We have observed comparable growth of mutant transformed with PiMgT1 with the WT when grown on 10 μM, 100 μM, 1 mM, 4 mM, and 10 mM Mg. Importantly, mutant transformed with empty vector and mutant transformed with PiMgT2 did not show growth on these Mg concentration (Figures 6A–E). However, a growth was observed in case of all the four strains when the concentration was increased upto 100 mM Mg (Figure 6F). We have also observed growth in case of all the strains when grown on YPD containing 4 mM Mg which was used as a control (Figure 6G).

### Cobalt Resistance Assay

It has been reported that CorA family genes helps the cells in Mg uptake but also provides resistance against heavy metal like cobalt. Hence, to confirm the cobalt resistance capacity of both the genes, cobalt resistance assay was performed. Each complemented strain were grown on two different concentration of cobalt i.e., 100 and 500 μM and fixed concentration of MgCl_2_ (100 mM). We found that both WT and mutant transformed with PiMgT1 were able to grow on 100 and 500 μM CoCl_2_, whereas, mutant transformed with the vector only and with PiMgT2 did not show any growth on any of the concentration of CoCl_2_ (Figures 6H,I).

### Growth Assay and Functional Characterization

**FIGURE 7:**
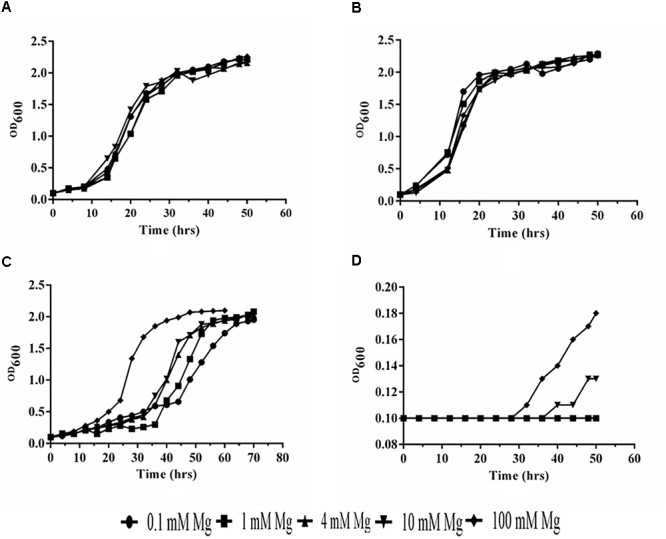
Analysis of growth. **(A)** Mutant transformed with PiMgT1 **(B)** WT **(C)** mutant transformed with the PiMgT2 **(D)** Mutant only.

We have observed that PiMgT1 restored the growth of mutant and showed comparable growth with that of WT when grown at low concentration of Mg (0.1, 1, 4, 10, and 100 mM) (Figures 7A,B). We have also observed a slow growth in case of mutant transformed with the PiMgT2 as compared to the WT and mutant transformed with the PiMgT1 (Figure 7C). Our result indicated that mutant was not able to grow at low Mg concentration (0.1, 1, 4, and 10 mM) but showed very slow growth when standard media was supplemented with 100 mM Mg (Figure 7D).

**FIGURE 8:**
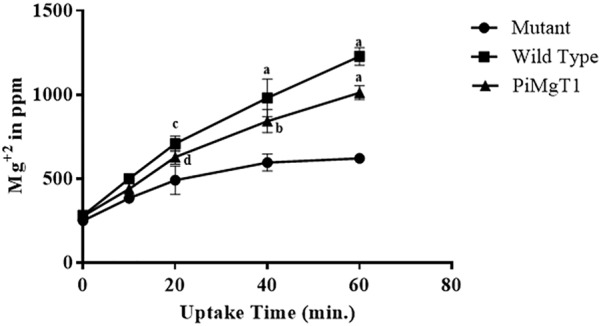
Uptake of Mg. Uptake of Mg by mutant, WT, and mutant complemented with *PiMgT1.* Significant differences (*p*-value) were calculated with respect to mutant using two way ANOVA ^d^*p* ≤ 0.05, ^c^*p* ≤ 0.01, ^b^*p* ≤ 0.005, ^a^*p* ≤ 0.001.

In case of mutant complemented with the PiMgT1, total intracellular Mg content was found to be two fold after 20 min as compared to the mutant and this difference was found to be statistically significant (*p* ≤ 0.05). We have also found more than four fold increase in the Mg contents of complemented mutant after 1 h as compared to the mutant (Figure 8).

## Discussion

Endophytic association of *P. indica* has shown as beneficial tool for the host plant to survive under abiotic and biotic stresses (Trivedi et al., 2013; Jogawat et al., 2016; Narayan et al., 2017). Additionally, *P. indica* can grow axenically, can colonized dicots, monocots and other medicinal plants which cannot be colonized by the AMF, therefore this fungus has been termed as plant probiotic (Aschheim et al., 2005).

Though reports are available which shows that AMF benefited the host plant during colonization under Mg limited conditions (Wu et al., 2006; Doubková et al., 2012; Zhang et al., 2015). However, no information is available related to the functional characterization of the Mg transporter of the AMF. In this study, Mg transporter from *P. indica* has been identified, isolated, and functionally characterized and also the role of *P. indica* in the plant growth and development has been investigated. When plants colonized with the *P. indica* and grown under Mg-limited condition, were found to be healthy and greener as compared to the non-colonized plants which showed retarded growth. Zhang et al. (2015) have reported that high concentration of Mg results in the low colonization of the AMF, in contrast to this report, we have found 70% colonization at 15 dpi. at all concentrations of Mg including 25 mM Mg, indicating that colonization of *P. indica* is independent of concentration of Mg. In order to check the impact of *P. indica* on plants, grown under Mg-limited condition, growth parameters were measured. We found that *P. indica* colonized plants have higher biomass, more number of leaves as compared to the non- colonized plants when grown at 4 mM Mg. Similarly, *P. indica* colonized plants found to have high amount of chlorophyll a, b and total chlorophyll at 4 mM Mg concentration as compared non- colonized plants. Similar observations were made by Zhang et al. (2015). They have found that when citrus plant colonized with the AMF *Funneliformis mosseae* under Mg-limited condition, AMF inoculation results in the increased biomass and chlorophyll contents of the inoculated plants as compared to the non-inoculated plants. Thus this study support our data. We proposed that, the long roots of non-colonized plants were due to the inaccessibility of Mg to the plants. Whereas, under the presence of *P. indica* the root length was found to be shorter which indicates that fungus is helping plants in the uptake of Mg from media. In case of colonized plants, leaves numbers were found to be high at 4 mM Mg as compared to the non-colonized plants. This indicates that the presence of an optimal amount i.e., 4 mM Mg in the medium enhances the leaves number. However, 25 mM of Mg found to be toxic to the plants as they found to be short, less number of leaves, chlorophyll contents with less biomass as compared to the plants grown at 4 mM Mg.

Further, to identify Mg transporter of *P. indica*, we have performed BLAST analysis by using Mg transporter of *S. cerevisiae* (ALR1-p) as a template against the *P. indica* genome. Our BLASTP analysis revealed that *P. indica* genome contains two putative Mg transporter showing significant degree of identity with the *S. cerevisiae* Mg transporter. Similarly, several studies proved that CorA-like proteins may represent the main transport systems in other organisms including eukaryotes like yeast, animals, as well as plants (Bui et al., 1999; Schock et al., 2000; Graschopf et al., 2001; Li et al., 2001; Kolisek et al., 2003). Our bioinformatics analysis revealed that both the putative Mg transporter of *P. indica* belongs to PIRI_contig_0025 (Accession no CCA67920.1 and CCA67912.1) and it falls under the range of MIT superfamily (Knoop et al., 2005). Further, we observed significant similarities in homology and phylogeny of deduced amino acid sequence of both putative Mg transporters (*PiMgT1* and *PiMgT2*) of *P. indica* with known Mg transporter of prokaryotes, fungi, higher plants and animals. Phylogenetic analysis with diverse group of organisms indicates that both the putative Mg transporters are more closely related to high-affinity Mg transporter of fungi and is far distinct from plants, animals and prokaryotes. Both the Mg transporters of *P. indica* were clustered among fungal CorA family members nearer to basidiomycetes. We observed that PiMgT1 have homology with other fungi, animals, plants and bacteria. When CLUSTALW analysis with the other group of fungus was performed, we found the presence of signature motif GMN (Glycine, Methionine, and Asparagine, responsible for the Mg transportation) in Mg transporter of *P. indica*, similarly, presence of GMN motif was also reported in case of ALR proteins of *S. cerevisiae*, therefore, this study support our data (MacDiarmid and Gardner, 1998).

For complementation assay, we have used Mg double mutant of *S. cerevisiae* lacking Mg transporter gene (Li et al., 2001). CM66 mutant was individually transformed with both transporters *PiMgT1* and *PiMgT2*. Spot assay confirmed that PiMgT1 complemented the mutant the most than that of PiMgT2. Further, WT and mutant transformed with PiMgT1 have shown typical diauxic growth in comparison to mutant transformed with the PiMgT2. Interestingly, only PiMgT1 complemented strain showed similar growth pattern like WT whereas, mutant transformed with PiMgT2 showed retarded growth. Hence, these results signify that PiMgT1 restores growth defects. Further, we observed that only complemented PiMgT1 and WT were able to grow on the media containing 10 μM Mg whereas, complemented PiMgT2 and mutant was not be able to grow at the same concentration. When concentration of Mg in the media increased from 10 to 100 mM Mg, both PiMgT2 and mutant showed good growth. A similar kind of study was reported in case of *A. thaliana* Mg transporter, MRS2/MGT family proteins and transformed in CM66 mutant, it showed similar kind of growth pattern and thus support our data (Saito et al., 2013).

Our bioinformatics data also showed that both putative Mg transporters belongs to CorA family, CorA family members are responsible for providing resistance against cobalt to the cells as well as for transportation of Mg across the membrane. Hence, we also performed cobalt resistance assay and found very interesting result that upto 500 μM cobalt concentration only WT and PiMgT1 was able to grow whereas, mutant and mutant transformed with PiMgT2 did not grow on the same concentration. Hence, it was confirmed that PiMgT1 belongs to the CorA family.

We have performed Mg uptake assay using WT, complemented mutant with PiMgT1 and mutant. Our ED-XRF analysis showed that amount of Mg content in case of WT and complemented mutant, increased with the time and both the strains showed the same pattern of Mg uptake whereas, mutant showed the negligible amount of Mg content as compared to WT and complemented strains. Hence, our ED-XRF analysis confirmed that PiMgT1 is helping the mutant to restore the Mg uptake ability. Similarly, Li et al. (2001) found an increase in the cellular Mg content after 60-min of uptake period in case of mutant transformed with plant Mg transporter AtMGT10, thus supporting our data.

Our study confirmed that *P. indica* has plant growth promoting activities and facilitating the plants to attain higher biomass, number of leaves as well as increased chlorophyll contents during Mg deficiency. The current study highlights the importance of *P. indica* Mg transporter (*PiMgT1*) for the improvement of Mg nutrition to the host plants when grown under Mg deprived condition. As *P. indica* has been termed as a “Plant Probiotic,” therefore, we propose that *P. indica* could be a better candidate for sustainable agriculture system to improve crop production in Mg-deficient agriculture field. Our present study will open new vista in order to apply *P. indica* as bio-fertilizer in the Mg deprived field to improve crop yield.

## Author Contributions

DP designed and performed the experiments. MB, NV, AS, and ON performed the experiments. AJ and MD provided chemicals. DP, AJ, and MD wrote the manuscript. MD and AJ initiated and supervised the project.

## Conflict of Interest Statement

The authors declare that the research was conducted in the absence of any commercial or financial relationships that could be construed as a potential conflict of interest.

## Footnotes

1 http://genes.mit.edu/GENSCAN.html

2 http://web.expasy.org/translate/

3 http://www.ncbi.nlm.nih.gov/BLAST/

4 http://www.expasy.ch/prosite/

5 http://www.ncbi.nlm.nih.gov

6 https://www.ebi.ac.uk/Tools/msa/clustalo/

7 http://www.megasoftware.net/
