# Supplementary material for: Functional Characterization of a Magnesium Transporter of Root Endophytic Fungus Piriformospora indica
Source: Front Microbiol. 2019 Jan 9;9:3231. doi: 10.3389/fmicb.2018.03231 (PMC6333687; doi:10.3389/fmicb.2018.03231)
Supplement: Supplementary file 1 [file Data_Sheet_1.doc]

**Supplementary Information**

**Functional characterization of a magnesium transporter of root endophytic fungus *Piriformospora indica***

**Durga Prasad^1^, Nidhi Verma^1^, Madhunita Bakshi^1^, Om Prakash Narayan^1^, Alok Kumar Singh^1^, Meenakshi Dua^2^ and Atul Kumar Johri^1*^**

**^1^School of Life Sciences, ^2^School of Environmental Sciences, Jawaharlal Nehru University, New Delhi-110067, India.**

***Corresponding author:akjohri14@yahoo.com, meenakshi72@hotmail.com**

**Table S1**: **Primers used in this study.**

| **Name** | **Forward sequence (5’…3’)** | **Reverse sequence (5’…3’)** | | **Purpose** |
| --- | --- | --- | --- | --- |
| **Cloning of *PiMgT1*** | | | | |
| PiMgT1 | GGGGTACCATGGAAAGTTTAGTGACCCCGA | GCTCTAGACCCTGCCTTCTTCGCTTC | | Cloning of *PiMgT1* |
| **Cloning of *PiMgT2*** | | | | |
| PiMgT2 | CGGGATCCATGCCCCGCGAAGACAGTGAA | GCTCTAGACTTCTTGCTGTCCTGTAGCACC | Cloning of *PiMgT2* | |
| PYES2_seq1 | GCAAAAACTGCATAACCACTTTAAC | | Sequencing of *PiMgT1* | |
| MgT1_seq1 | CACACGCGCACTTTCATGATG | |
| MgT1_seq2 | GCTTCATCCACTATCAATAGAGG | |
| MgT1_seq3 | GTAGCCAAGCTAGAGGCTCTG | |
| MgT1_seq4 | GTAGATTCGACCTAGAAAGG | |
| PYES2_seq1 | GCAAAAACTGCATAACCACTTTAAC | | Sequencing of *PiMgT2* | |
| MgT2_seq1 | CTTTCAACCCCCTTGGGCCC | |
| MgT2_seq2 | CTCCTCTGGTTCGCATATC | |
| MgT2_seq3 | CATCCAGCGACCCTTCGATG | |
| MgT2_seq4 | GCAGAGAATCTCATCAACTAC | |

**Table S2. Members of Mg transporter homologs proteins used for phylogenetic tree analysis.**

| **Sr. No.** | **Organism** | **Mg^+2^ transporter** | **GenBank Acc. No.** | **Division** |
| --- | --- | --- | --- | --- |
|  | *Piriformospora indica* | PiMgT1 | CCA67920.1 | Fungi |
|  | *Piriformospora indica* | PiMgT2 | CCA67912.1 | Fungi |
|  | *Hypsizygus marmoreus* | HmMgT | KYQ46102.1 | Fungi |
|  | *Ustilaginoidea virens* | UvMgATPase | KDB10842.1 | Fungi |
|  | *Mycena chlorophos* | McMgT | GAT55268.1 | Fungi |
|  | *Magnaporthe oryzae* | MoMRS2 | ELQ36153.1 | Fungi |
|  | *Aspergillus oryzae* | AoMRS2 | EIT82504.1 | Fungi |
|  | *Cryptococcus neoformans* | CnMgT | AFR96726.1 | Fungi |
|  | *Rhizoctonia solani* | RsMRS2 | CUA75477.1 | Fungi |
|  | *Rhizoctonia solani* | RsMgT | EUC67427.1 | Fungi |
|  | *Pleurotus ostreatus* | PoMgT1 | KDQ31457.1 | Fungi |
|  | *Grifola frondosa* | GfMgT | OBZ68633.1 | Fungi |
|  | *Laccaria bicolor* | LbMgT | XP_001874189.1 | Fungi |
|  | *Laccaria bicolor* | LbATPase | XP_001873732.1 | Fungi |
|  | *Saccharomyces cerevisiae* | ScARL1 | Q08269.1 | Fungi |
|  | *Saccharomyces cerevisiae* | ScMgT | EDN59845.1 | Fungi |
|  | *Saccharomyces cerevisiae* | ScARL2 | P43553.1 | Fungi |
|  | *Saccharomyces cerevisiae* | ScMRS2 | Q01926.2 | Fungi |
|  | *Arabidopsis thaliana* | AtMHX | AAF14229.1 | Plants |
|  | *Triticum aestivam* | TaMgATPase | P83970.1 | Plants |
|  | *Nicotiana tabacum* | NtMHX | XP_016511514.1 | Plants |
|  | *Brassica napus* | BnMHX | XP_013691805.1 | Plants |
|  | *Medicago truncatula* | MtMHX | XP_003591515.1 | Plants |
|  | *Solanum lycopersicum* | SlMHX | NP_001275917.1 | Plants |
|  | *Oryza sativa* | OsMRS2-7 | BAD82756.1 | Plants |
|  | *Zea mays* | ZmMRS2-G | XP_008658098.1 | Plants |
|  | *Zea mays* | ZmMRS2-H | XP_008650823.1 | Plants |
|  | *Zea mays* | ZmMRS2-D | XP_008663199.1 | Plants |
|  | *Sinorhizobium meliloti* | SmMgT | AEG03959.1 | Bacteria |
|  | *Clostridium lentocellum* | ClMgT | ADZ83024.1 | Bacteria |
|  | *Salmonella enteric* | SeMgATPase | AH1058 | Bacteria |
|  | *Escherichia coli* | EcMgATPase | D86122 | Bacteria |
|  | *Clostridium botulinum* | CbMgT | EDS78697.1 | Bacteria |
|  | *Cyanobacterium aponium* | ClMgT | ADZ83024.1 | Bacteria |
|  | *Xanthomonas oryzae* | XoMgT | KOR48351.1 | Bacteria |
|  | *Homo sapiens* | HmMRS2 | NP_065713.1 | Mammals |
|  | *Rattus norvegicus* | RnMRS2 | NP_076491.1 | Mammals |
|  | *Mus musculus* | MmMRS2 | NP_001013407.2 | Mammals |

Start codon

**ATG**GAAAGTTTAGTGACCCCGAAGCATCCTCAGCGTCGACAGCGAGAGAGCGAAGACGGGTCAGACGATCCCGACGACTTTGACGCACCTCCGGACGTGAGCGCAGCGTATGGCCTTTCACCGCCGATAGAGGTCACCAGTCCCACGCACGAAACGGGACATGTCACACCAGGACACATTGTCGGCAGGCCAATTCACGAGTCTCCAACACACGCGCACTTTCATGATGTACACGATACAAATGGTTTACCGCCACCAAACCTCATGAGCATGGATAACGCGCGCTTTGGACGTGATATACAGGCTATCGAAGCTCAACACAGCAGCGAGACGCTCGTCGAACCGTTTGTTCGCGCGCCAGGAGCAGAACCAGGCCTAGATCCAAGAAGAGAATCTACAAATAGGGCATACGCTCACATTAAAGAAGATTGCGAGATTGAGGTCGTGGATTACAGCGAGGACCGTGTTCGGTTTCAACAATTCCACAATGAATCGTTTATCGCCTTTCTCAGGGGGTGCGACCGTCAGCCTCCTATGAAAGTTCGATGGATCAATATAGCTGGTATATCATGGGACGTCATAAGTGCCCTAGCCCTAAAGTACCAGCTTCATCCACTATCAATAGAGGATATTATGCACAGTGGCCCGACCACACGCTCAAAAGCAGACTACTACCAACGACATTTGTTTTTGCATGTCCTATGCCATGCCTTGCACTCGAAAAGATCGTCTCCTCTCGACGCAAAGTACCTCGCCGAAGAAGCCGATGCAGCAACAACCTCAGAGTCTGAAGACTCGGAGGAGACCACGGTTACGGACGGCGGAACTGAGGCTGAAGAGGAAGAGTCGAATCGCCGTGCTTTGCTTCGGCGCGTCACAAGACGTGGTAAGAGACGGCGGACAAAGTCTGTAGACACCGAAGCGTCGGCGGGGAATAGCCAGAGTCGCATGGGTAGCATGCGCTGGTCCGAGGCAGTTGGACTGCTAAGTCCGGAAGGCCGTCGGCGGCAGGCACGGGTAGCCAAGCTAGAGGCTCTGAAGAAACGAGACCGAGTGCGGGTGCTAGTCTCGAATGCATTCTTTTTTCTCTTTAAAGATGGGACGGTCATCTCGTTACATCAGGGCGACCGTGCGTTTGGAAATCCAATCTATAATCGACTCCGGCATTCCGACACTGTGCTGCGAAAGGATCCAGAGGGGTCTCTCCTTCTCCAATCGCTACTTGATCTCATTGTAGATCAGATGTTGGATGTCATAGACAAATATGGCGATAAAATTAATGAGAGTGAGACTCATGTCTTACTTAAACCCAATATGGATGTAGTTCGAAGTCTACACATTCTATCAGCAGACTTGACCTTGAGGAAGCGGACGATGCAACCTCTCAAGACGCTCATCTATGGGCTTCGTAGATTCGACCTAGAAAGGACAGCAGCAGCGCTCGCCGTTGCTGGAATTCCCCAGGGCCAGGTGCAGGGGTATATGAGCCCAAAGACGAAAGTCTACCTGGCTGATATTAGCGACCATTTAGAAACGATCACATCCTCTCTCGAGCAATTCAACACTATGACCGACAACCTGATCGATTTCGTCTTTAATATGAATGCGCATGCGACGAATAACCAGATGAGACGAATGACCATTCTGACGCTCATTTTCCTGCCCATAACGGCCACGACTGGATACTTTGGGATGAATTTCGCGACGATGCCGTCTGTGCAAGAGCATAGCGAGGCCATGTTTTGGCAGATTATCCTACCTGTCATGGTGGTGGTGGTGACGTGGGCATTATGGACGGACATTTCGAGGGTGTTCAAGATGTTTGGGCGACTGCGGTTGCTCAAAGGCGTCGAGGAGCGACAAAAGACGAAAGCACGGGATGCAAAGCGAATTGACCGGAAGCGACGGCAAACGATGGAAGCGAAGAAGGCAGGG**TGA**

**Stop codon**

**Figure S1: cDNA sequence of putative *PiMgT1***

Start

**ATG**CCCCGCGAAGACAGTGAAGGAAGCAGCGGCGGGTCCATCACCTCCGATGAGGAAGAAGTTCCAACACACATGGCCGTCGCGTCAGAGGCAGCGTCTGCAACAGTCGCCGGTACCGTTCGTGGTAGTCCAGCTCTTCAGAAACTCCGTAGTAGCGTCAACAAGGTTATTCGTATGAACAAGATCCAATCCGCTTTCAACCCCCTTGGGCCCGGCTCCGAGCCCGGGATAGATCCAAGAAAGCCAGGGGTCGACGCTGCCTACGGGCATATTCGGGAACGTTGCGAGATTGAGATTATTGACTACAGCGCGAGCAACTATGTCATTAACCAATATAACAACGAGGAATTTGTCAATCTCATGCAGTCTACGGGTGGAACGCGTCCCGGATGGGCCAAAGTCCGGTGGATTAACATTGGCGGTACAAGCTGGGATGTTCTTTCCAAGCTCGCAATTGCATACAAGCTTCATCCACTCACATTGGAGAACATTGTCGCCGGCATGCCCCAAACGTCCCGCAGCAGAGCCGATTATTATACCAACCATCTTTTCTTGCACGTCATGTGAAGCCTAGAAGAAAAGGAGAGTTCTGCTTCTCCTCTGGTTCGCATATCCAATCCAGCTCGGAGTCGCTCGCCCGCTCAGATGAATCACCATGAGCACACGCACGGAGGTGCAGCTGAAAAGGCGCCATCACTTCGCCGACGTAAAGGCGTAGCGCCAGCACCAGATATTGAAATGGGTAGACCTCAACCCAGGAAGGAGAAAAAGCGCAAGTCGCGCATGACGACCCTAGACATTCGCAATCAGAAGAATGAGAGTCTCAAGCGCGCCGAAGAACTCAACGAGGCCCTTCGAGATACCATGTTCACCGTCAAGGTGACGACCAAGAGTCTATTCTTGTACCTTCTACGGGATGGAACGGTCATCTCTGTCCATCAAGCTGACAGAGGCTTTGGCGACCCGATTTATCGACGATTGCAGTCACCGACCACGGTTCTGCGAGCATCCAGCGACCCTTCGATGCTTTTGGAGGCACTTTTGGATCTGGTCGTCGACCGTGCGCTGGACATTGTGGATCGGTACCATGAAAACATTCTGGCCGTCGAGAGGAGCATCCTGCTCAACCCCAAGATGGTTGCAATTCGTCAACTTCACTTTTTCTCGCGCGACCTGACGGCCCGCCGACGTGAGCTGGACCCAGTCAAGACGCTCATCTATGGTTTGCGCCGTTATGACAAGGAGCGCTGTGCTGCCGTCGCCGAGTCTGCTGGCATTCTTGTCGAGGGCGAGGCGGTCCAAGGCTTCCTTAGCCACAAGGCCAAAATCTATCTCGCGGATGTCCACGAACACTGCGAATGGATATTGACCTCATTTGAGATGCAAGCTGACATAGCAGAGAATCTCATCAACTACACATTCAATACCATTGCATACGAGACGAACAATACGATGCGCACGCTTACACTGGCCACGATCATCTTCCTGCCGCTTACGTTTTTGTCTGGGTACTTTGGAATGAATTTCGAAGTCTTTGGCGGAATCCAGCATTCAGATGCATACTTCTGGGAGATTGCGGCACCGGTGTTTGTGGCGACGGTCATCATCTTCCTATGGCACGATTTGCGAAGGATGTGGCATTTCCTCAAGAAGAAGACGTTCTTGCATCGATTCGTACGTACTCGCCTCCTGCGCTGGTTTTCGAGTGTTCACCCCAAAATCTCTTCGACCCATTCATTTGCTGCGCATAACATTCATATGGAAAACTCGCTTCTTCGACATTTGACATGGTTTGCAAACGACGAATATAAACATCTGTATTCGCCACCCACTCAACGGCATGTTGTGGCATCGGATGCCACCCCTTCGAACCCCTGGTTGCGTCACGCCTCGCATTTGGTGCTACAGGACAGCAAGAAG**TAG**

Stop

**Figure S2: cDNA sequence of Putative *PiMgT2***
